# Taking Stock of Tobacco Control Program and Policy Science and Impact in the United States

**Published:** 2017-09-15

**Authors:** Matthew C Farrelly, Frank J Chaloupka, Carla J Berg, Sherry L Emery, Lisa Henriksen, Pamela Ling, Scott J Leischow, Douglas A Luke, Michelle C Kegler, Shu-Hong Zhu, Elizabeth M Ginexi

**Affiliations:** 1 Center for Health Policy Science and Tobacco Research, RTI International, 3040 E. Cornwallis Road, P.O. Box 12194, Research Triangle Park, NC 27709, United States; 2 Health Policy Center, Institute for Health Research and Policy, University of Illinois at Chicago, 444 Westside Research Office Bldg. 1747 West Roosevelt Road Chicago, IL 60608, United States; 3 Department of Behavioral Sciences and Health Education, Rollins School of Public Health, Emory University, 1518 Clifton Road NE, Atlanta, GA 30322, United States; 4 NORC at the University of Chicago, 55 East Monroe Street, 30th Floor Chicago, IL 60603 United States; 5 Stanford Prevention Research Center, Stanford University School of Medicine, 1070 Arastradero Road, Suite 353, Palo Alto, CA 94304, United States; 6 Center for Tobacco Control Research and Education and Division of General Internal Medicine. University of California San Francisco, 530 Parnassus Avenue, Suite 366, San Francisco, CA 94143, United States; 7 Public Health Program, College of Health Solutions, Arizona State University, 550 North 3rd Street, Room 512E Phoenix, Arizona 85004, United States; 8 George Warren Brown School of Social Work, Washington University in St. Louis, 700 Rosedale Ave, St. Louis, MO 63112-1408, United States; 9 Department of Family Medicine and Public Health, University of California, San Diego, 9500 Gilman Drive #0905, La Jolla, CA 92093, United States; 10 National Cancer Institute, National Institutes of Health, 31 Center Dr., Room B1C19, Bethesda, MD 20892, United States

**Keywords:** State, Community, Tobacco control, Policy, Surveillance

## Abstract

The 60% decline in the prevalence of cigarette smoking among U.S. adults over the past 50 years represents a significant public health achievement. This decline was steered in part by national, state, and local tobacco control programs and policies, such as public education campaigns; widespread smoke-free air laws; higher cigarette prices that have been driven by large increases in federal, state, and local cigarette excise taxes; and other tobacco control policy and systems-level changes that discourage smoking. Using the MPOWER framework informed by the Centers for Disease Control and Prevention (CDC) Office on Smoking and Health and the World Health Organization (WHO), this paper reviews these accomplishments and identifies gaps in tobacco control policy implementation and additional research needed to extend these historic successes.

## Introduction

Over the past five decades, the prevalence of cigarette smoking among U.S. adults has decreased by more than half from 42% in 1965 to 16.8% in 2014 [1]. Per capita cigarette consumption has declined by 69% from 2,702 packs in 1965 to 845 in 2014. The first report on smoking and health from the Surgeon General in 1964 [2] started a process of education and social change that has dramatically altered how Americans now view smoking. Tobacco control efforts are estimated to have prevented 8 million premature deaths and to have extended mean life span by 19 to 20 years in the United States between 1964 and 2012 [3]. Mass media campaigns and tobacco control policies have been key drivers behind this significant population-level change. These successful efforts are consistent with Frieden’s public health pyramid, in which state and community tobacco control efforts aim to enact policies that have significant reach and change the context in which decisions about smoking are made [4].

The 2009 Family Smoking Prevention and Tobacco Control Act (Tobacco Control Act) ushered in a new set of tools for reducing tobacco use. The Tobacco Control Act gave the Food and Drug Administration (FDA) the authority to regulate tobacco products and enables state and local governments to regulate the time, place, and manner of tobacco advertising by removing federal preemptions. Key to FDA’s authority is the ability to set product standards that may make tobacco products less attractive and/or addictive. Although this new authority holds great promise for further reducing tobacco use, it largely focuses on product regulation and does not include most tobacco control policies and programs at state and local levels that have been extremely successful in driving the declines in cigarette smoking [5]. FDA regulatory authority alone is unlikely to be sufficient to end the tobacco epidemic, in part because many of the most effective tobacco control measures are outside of FDA’s authority.

Although tobacco control represents an important public health achievement, many challenges remain to further decrease population-level tobacco use, including continued vigorous opposition of the tobacco industry in the United States and around the world [6–9]. Approximately one in six adults (16.8%) 40 million Americans are current cigarette smokers [1]. Significant disparities in smoking prevalence exist, based on income, education, race/ethnicity, and other factors. Additionally, secondhand smoke (SHS) exposure is still a common risk, particularly in children, non-Hispanic blacks, and low-income populations. Cigarette smoking and exposure to tobacco smoke cause about 480,000 premature deaths each year in the United States [5]. Of these premature deaths, about 36% are from cancer, 39% are from heart disease and stroke, and 24% are from lung disease [5].

Inhalation of smoke from burning tobacco is still the most deadly risk behavior in the United States. However, consumers are now bombarded with many other non-cigarette tobacco products, including cigars, little cigars, and cigarillos; hookah; various smokeless tobacco products; and a heterogeneous collection of electronic nicotine delivery systems (ENDS) and vaporizers. Many of the non-cigarette tobacco products particularly appeal to youth and young adults because they can be used to inhale mixtures that contain nicotine, chemical flavorings, or other substances, such as cannabis or cannabis oil. The concerns about these products include misperceptions of potential health risks [10–14], use as an alternative to smoking cessation [15], and facilitation of polytobacco or polysubstance use [13,16–19]. For example, many young adults are using little cigars and cigarillos either alone or together with marijuana by “topping off” their blunt with regular cigarettes [20]. Young adults are also using ENDS to vaporize cannabis in the form of highly concentrated liquid hash oil or dried cannabis buds or leaves [21]. The population-level impact of ENDS on smoking rates is still uncertain. Some studies have shown that ENDS have the potential to improve population-level smoking rates [22], whereas others note that they are not yet widely used and may only have a meaningful impact on smoking cessation if the technology improves [23]. In addition, emerging evidence indicates that ENDS may encourage youth smoking by introducing youth to nicotine [24–27]. Most of these new products are advertised with innovative and targeted marketing, promotion, and product placements using social media channels that users can access through smartphones. This makes for a much more complex and rapidly changing landscape within which to study or to implement existing or new tobacco control policies. Innovative policy research that is practical, nimble, creative, and highly responsive to these emerging new product domains and user populations will be necessary to strengthen and reinforce antitobacco social norms across diverse communities and to counteract pro-tobacco marketing.

In this paper, we review key public health and research accomplishments to date and identify the next steps for research within each area of focus at the state and community levels using the framework identified by the Centers for Disease Control and Prevention (CDC) Office on Smoking and Health and the World Health Organization (WHO). The areas of programming and research focus have been represented by the acronym MPOWER: Monitor tobacco use and prevention policies; Protect people from tobacco smoke; Offer help to quit tobacco use; Warn about the dangers of tobacco; Enforce bans on tobacco advertising, promotion, and sponsorship; and Raise taxes on tobacco. We briefly detail the progress made so far within each of the six areas of tobacco control policy and program science, discuss some dissemination and implementation opportunities, and conclude the paper with a few overarching recommendations regarding directions for future research. We do not address strategies that fall primarily within FDA’s purview, such as tobacco package warning labels.

### Monitor tobacco use and prevention policies

#### Primary purpose

Tobacco surveillance is critical to efforts to reduce the burden of tobacco use on individuals and society. Although accurate surveillance of population-level use patterns is crucial, it is equally important to have comprehensive surveillance of the tobacco control policies and interventions that shape tobacco use.

#### Key accomplishments

Tobacco surveillance in the United States has a long history and yields high-quality data on cigarette smoking and many federal, state, and local policies. The current tobacco surveillance systems capture the prevalence and quantity of cigarette use and the prevalence of many non-cigarette tobacco products, including cigars, smokeless tobacco, hookah, and e-cigarettes. Existing surveillance systems capture the use of these products among youth and adults on an annual basis. In addition, the Population Assessment of Tobacco and Health study is a longitudinal household survey that monitors tobacco use among a large cohort of youth and adults.

#### Gaps in implementation

Existing tobacco surveillance systems in the United States have many strengths but also several limitations. The accuracy of cigarette and non-cigarette tobacco product use measures is limited due to several key problems. First, tobacco product use patterns are changing. Daily cigarette smoking is declining [28], while nondaily and light smoking is increasing [29]. As many smokers report nondaily cigarette use for sustained periods [30,31], existing measures need to be updated to capture the range of current and former tobacco use patterns across systems [32,33]. The intensity and frequency of non-cigarette tobacco product use are not systematically and accurately captured. This is not surprising given the diversity of product package sizes and tobacco use patterns. Given that new products (e.g., ENDS) and new use patterns are emerging (such as increases in intermittent smoking, multiproduct use, and co-use with marijuana in combusted and vaporized form), relevant, valid, and reliable survey items are needed to capture prevalence, use patterns, perceptions, and their correlates accurately. Due to the variation in harm posed by the current range of products, assessing population health effects is dependent on understanding the changing patterns of tobacco product use [34]. In addition to tobacco use, there are ongoing critical needs for surveillance of state and local policy adoption and implementation, tobacco industry marketing efforts, tax avoidance/evasion, changes in the overall tobacco market, and public attitudes about tobacco as reflected in traditional and new social media. New communication technologies, such as mobile and social media, make using traditional modes of survey research (e.g., phone surveys) problematic but also present new opportunities to recruit hard-to-reach populations (e.g., lesbian, gay, bisexual, and transgender [LGBT] people; pregnant women; racial/ethnic minorities), reduce burden on respondents, and rapidly collect high-quality data that are accessible to researchers and policy makers.

Despite calls for establishing and maintaining monitoring systems for policies, tobacco marketing, and public sentiment about tobacco issues in social media, including a national system for local tobacco control ordinance surveillance and a comprehensive state tobacco control program monitoring system [35], only modest progress has been made on this front.

#### Existing evidence and needed research

The current tobacco surveillance environment illuminates challenges that limit data quality (e.g., coverage, nonresponse, and measurement error), efficiency, and timeliness. Survey questions, in particular, require continuous assessment for their appropriateness across diverse sociodemographic groups (e.g., age, race/ethnicity) and due to changes in respondent interpretation over time. For example, the criterion of having smoked more than 100 cigarettes in one’s lifetime to be considered an ever smoker, dates back to the 1950s [36] and may need to be revisited for contemporary populations. Existing surveillance systems are also limited in their ability to collect measures on a continuous or frequent basis (i.e., rapid succession of longitudinal measurements).

Changes in technology have contributed to declining success with surveys, including lower response rates and other sources of sampling bias [37,38]. Additional research is needed to identify how to improve existing systems to provide more accurate data using updated measures and data capture methods.

### Protect people from tobacco smoke

#### Primary purpose

The 1972 U.S. Surgeon General’s report was the first Surgeon General’s report to highlight SHS exposure as potentially harmful to the health of nonsmokers, although evidence of the dangers of exposure dates back to the 1960s [39]. A major tobacco control goal is the establishment of comprehensive smoke-free policies to provide protection from the harmful effects of SHS exposure [40]. A comprehensive policy bans smoking in all enclosed public places and workplaces, including bars, restaurants, and public transportation [41].

#### Key accomplishments

In 1964, few limits on smoking in public places existed. According to the American Nonsmokers’ Rights Foundation, as of January 1, 2015, 65% of the U.S. population lived in communities that banned smoking in the workplace, and 49% lived in places that also banned smoking in restaurants and bars. Twenty-six states, commonwealths, and U.S. territories and a long list of local municipalities have such laws [42]. Numerous public health benefits, including increased cessation attempts among current smokers and decreased tobacco use prevalence, initiation among young people, tobacco-related morbidity/mortality, and health care costs have resulted without adverse economic impacts on businesses [40]. However, SHS exposure is highest among children, African Americans, those living in poverty, and/or those living in rental properties [43]. Children’s primary source of SHS exposure is in the home [43].

More recently, laws mandating such policies in private vehicles or in multiunit housing (MUH) have been implemented in some nations, states, and cities [44]. For example, as of 2015, 8 U.S. states and territories prohibit smoking in cars with children (with varying age requirements) [45]. In addition, as of 2015, 16 municipalities in California have legislated policies requiring entire MUH complexes to be smoke-free, with an additional 27 California communities having some sort of restriction within MUH [46]. Most recently, the Department of Housing and Urban Development proposed a rule that would require each public housing authority to implement a policy prohibiting combusted tobacco products in all living units, indoor common areas in public housing, and in public housing authority administrative office buildings. In addition, increasing numbers of households are establishing voluntary smoke-free home and vehicle rules as norms about smoke-free environments continue to strengthen [47].

#### Gaps in implementation

Unfortunately, disparities exist in the adoption and implementation of smoke-free policies. Even in states with comprehensive smoke-free air laws, exemptions exist, leading to exposure to SHS in warehouses, family-based day care, small businesses, and hotels among others [48]. These locations tend to employ low-wage workers who themselves have disproportionately high smoking rates [48]. Southeastern U.S. states have lagged in adopting public smoke-free policies [49]. Many reasons seem to contribute to this [50], one of which may be the historical importance of tobacco agriculture in these states and the opposition of the tobacco industry [51–53]. Adapting smoke-free and/or tobacco-free policies to address non-cigarette tobacco products (e.g., little cigars, cigarillos, e-cigarettes, hookah) that are gaining in popularity is a new challenge [52,54]. In addition to limited data on health effects and youth appeal, these products may be used to circumvent smoke-free policies [42]. Many public policies do not specify that hookah, ENDS, or other noncombustible tobacco products are banned. Addressing SHS exposure from marijuana in public and private settings is another emerging issue as states increasingly consider enacting policies allowing for medicinal and/or recreational marijuana use.

#### Existing evidence and needed research

Research must be conducted to study the impact of expanded smoke-free policies that cover a greater range of settings (e.g., private homes and vehicles, MUH) and a greater diversity of tobacco products, as well as marijuana, particularly in relation to health outcomes, social norms regarding use, youth initiation, and use rates in general. Another gap involves identifying ways to promote support for tobacco-free policies. Media coverage and advocacy efforts in support of or in opposition to tobacco control policies have framed messages in relation to health, economic issues, youth prevention, and individual rights [55–60]. However, limited research has examined the persuasiveness of different messaging strategies, particularly in relation to emerging issues, such as alternative products or settings (e.g., vehicles, MUH), and their impact on populations with disproportionate SHS exposure. Research is also needed to examine novel messaging strategies to garner support for smoke-free laws and policies in contexts where policy adoption is lagging and among diverse populations.

### Offer help to quit tobacco use

#### Primary purpose

Smokers who quit substantially reduce their risk of disease and premature death [5]. Specifically, quitting smoking reduces the risk for lung and other cancers, heart disease and stroke, and chronic obstructive pulmonary disease [5,61,62]. Moreover, quitting before age 30 avoids most health consequences of smoking [62]. There is substantial evidence that behavioral therapy (e.g., counseling) and pharmacotherapy (e.g., nicotine replacement therapy, varenicline) alone and in combination increase the success of smoking cessation [63]. In the past 5 years, evidence has emerged and solidified that varenicline and combinations of various forms of nicotine replacement (e.g., patch and gum) are most effective and should be encouraged as first-line medications for smoking cessation [64–66]. While the evidence base is strong for cessation treatment, there is a pressing need to identify successful mechanisms that can increase consumer demand for effective treatment and further promote policy and system changes that have helped drive smoking cessation to date [67]. Disparities in cessation also need to be addressed. Specific populations, such as LGBT, American Indian/Alaska Native populations, young adults, low-income groups, and people living with HIV/AIDS or with mental health conditions, continue to have high rates of smoking [1]. Much remains to be done to increase smokers’ desire and action to quit smoking (e.g., making cigarettes more costly and less socially acceptable) and broadening the reach of effective interventions in underserved populations.

#### Key accomplishments

The decline in adult smoking prevalence over the past 50 years has been driven both by an increase in the rate of smoking cessation and a decrease in smoking initiation [67]. A complementary set of factors has helped propel the decline in smoking. Public education campaigns have increased awareness of the health consequences of smoking and SHS exposure, motivated smokers to quit, and influenced social norms about smoking. For example, exposure to recent state and national public education campaigns is associated with increases in the population-level prevalence of making quit attempts [68] and intentions to quit [69,70]. Public education and changes in social norms have also facilitated increases in federal, state, and local cigarette excise taxes and smoke-free public spaces that are also associated with increases in smoking cessation [71,72]. In recent years, community-based approaches like quitlines have been implemented in every state, and insurance coverage via the Affordable Care Act (ACA) has made it easier for smokers to use proven treatments [73,74]. Health care systems, including federally qualified health care systems and behavioral health programs, are being encouraged to prevent disease by helping more smokers quit. Because of the ACA, state Medicaid programs are required to include an FDA-approved tobacco cessation product [75]. In the individual and small group health insurance marketplaces, health status rating is no longer permitted, but tobacco use rating is permitted in most states [76,77]. This has raised questions about insurance affordability for tobacco users, as well as concerns that tobacco users in these marketplaces may attempt to conceal tobacco use from health care professionals [78]. Understanding the impact of ACA changes in the health insurance industry affecting tobacco users is a pressing area for future research. Given the rapid uptake of ENDS in the market, it is important to understand whether ENDS are helping smokers quit, retarding progress, or having no effect. A recent review concluded that there is not sufficient evidence that ENDS increase smoking cessation [63]. Importantly, ENDS are not one product, but rather they reflect a large evolving group of tobacco products, which makes research in this area quite complex.

#### Gaps in implementation

Despite the availability of multiple treatment options in the changing health care environment, only a minority of smokers use formal assistance to quit [79], and the rate of physicians advising smokers to quit has remained relatively unchanged from 2000 to 2010 [80–81]. Despite 40% of all births being financed by Medicaid and eligible for a generous tobacco cessation product benefit [82], OBGYNs often do not refer pregnant women for behavioral health interventions [83]. This represents a lost opportunity to expand the use of treatment options. Additionally, gaps in coverage of tobacco cessation products remain; for example, Medicaid recipients may face financial barriers, such as co-pays for tobacco cessation products, or only be offered minimum product options [75]. Funding for state quitlines has been reduced in many states in recent years [84], even as large-scale media campaigns by CDC encourage more people to quit by promoting the national quitline number. Although intensive media campaigns have significantly increased quit rates [69,70], most state tobacco control programs do not spend sufficient amounts to promote cessation [85]. Efforts to reach specific populations with high rates of smoking (e.g., LGBT, low income, persons with mental health conditions, American Indian/Alaska Natives, young adults) are needed to address disparities in cessation. Tobacco use in these populations may also not fit traditional smoking cessation protocols, with increasing rates of both light and non-daily smoking, use of non-cigarette tobacco products (e.g., iqmik), and co-use of cigarettes with marijuana (e.g., cigarillos or blunt wraps). For example, tobacco users who smoke mainly “blunts” may not even recognize their smoking as tobacco use or acknowledge a need to quit [86], and little is known about how to reach and treat users of cigars/cigarillos or co-users of tobacco and marijuana.

#### Existing evidence and needed research

A significant challenge is that quit attempt and annual cessation rates in the United States have not increased for two decades [87]. Several questions need answers to increase population quit attempt rates. First, how can state and community-oriented investigators identify opportunities presented by health systems change, such as the ACA, to significantly increase smoking cessation and utilization of evidence-based treatment through systems changes and other interventions? Second, what population-based efforts outside of the health care system (e.g., workplaces, social environments) can be leveraged to increase smoking cessation in the population? Third, what are the effects of new policies, such as taxes, smoke-free policies, and legalization of retail marijuana, on tobacco cessation? Fourth, how can new media be used most effectively to drive engagement with smoking cessation [88]? Fifth, what community approaches will increase equity in smoking cessation by engaging populations disproportionately affected by tobacco to foster decreased tobacco use? Sixth, are ENDS helping smokers quit [63]? Finally, research is needed to address the population impact of the promotion and use of new and evolving tobacco products, especially the increasing use of ENDS, and the impact of state and local policies on smoking cessation patterns at the population level.

### Warn about the dangers of tobacco use

#### Primary purpose

State-sponsored antitobacco programs historically have relied heavily upon mass media campaigns primarily paid television advertising to promote tobacco control messages, such as highlighting the dangers of smoking and SHS exposure, exposing deceptive industry marketing practices, and, to a lesser extent, building support for tobacco control policies.

#### Key accomplishments

Between 1990 and 2002, more than 30 U.S. states launched mass media campaigns, the majority of which were financed by cigarette excise taxes and/or the 1998 Master Settlement Agreement [89]. Sufficient evidence now has demonstrated that these state antitobacco communication campaigns have effectively influenced attitudes, beliefs, and population smoking behavior [70,90–93]. In addition, in 2012, CDC aired Tips From Former Smokers (Tips), the first federally funded, nationwide, paid-media tobacco education campaign in the United States, [94] and in 2014, FDA launched The Real Cost media initiative targeting youth [95]. Evidence to date indicates that these campaigns, which reach a wider audience than their state-sponsored counterparts, have been successful [69,96]. The successful national truth campaign [97–99], which debuted in 2000, re-launched in 2014 after a brief hiatus.

#### Gaps in implementation

Although the evidence base for antitobacco media campaigns is solid, funding for state campaigns has declined along with funding for state tobacco control programs down 38% from its peak of $750 million across all states in 2002 to $468 million in 2015 [100]. The ability of communication campaigns to prevent and reduce population smoking is further complicated by changes in media consumption patterns. Although television remains the most important platform for news and information, mobile technologies and social media have transformed not only how people are exposed to and interact with health-related information, [101] but also how we watch television [102]. The proliferation of programming and platforms has created very segmented audiences, with few topics or campaigns that achieve broad exposure or engagement.

#### Existing evidence and needed research

Research investigating several practical questions is needed to guide and inform antitobacco communication campaigns. First, a new conceptual framework is needed to define how people are exposed to messages in the context of the evolving media landscape. Research is needed not only to establish this paradigm, but also to set standards of methodological rigor for collecting, analyzing, and interpreting data from emerging media sources. Second, research should investigate the effectiveness of alternative and nontraditional media channels in reducing tobacco use. Third, another core strategy for tobacco control programs has been to engage in policy advocacy and media advocacy to gain earned media as a way to build support for tobacco control and policy initiatives. With the advent of social media, however, it is important to research how the relevant dialogue in newer channels, such as Twitter, Facebook, YouTube, blogs, and online comments to news articles influences support for policy [88]. Fourth, more research is indicated to help tobacco control programs to better address tobacco-related health disparities among priority populations, including young adults, racial/ethnic minorities, and the LGBT communities. These groups not only may be at higher risk for tobacco use than the general population [103–107], they also are more likely to use new media [108]. To stem the disproportionate impact of tobacco use among priority populations, it will be essential for antitobacco advocates to identify subpopulations at increased risk for tobacco use and reach them with targeted messages through their preferred media channels.

### Enforce bans on advertising, promotion, and sponsorship

#### Primary purpose

Many forms of tobacco marketing contribute to experimentation with tobacco products, increase consumption, discourage quitting, and encourage relapse, but this section focuses on the retail environment because it is the least regulated marketing channel, and because this is where the tobacco industry spends nearly all its annual marketing budget (91% of the $9.5 billion in 2013) and where the majority of exposure to tobacco marketing occurs [109–111]. Given First Amendment constraints on banning advertisements [112], state and local policies have aimed to reduce product availability; increase prices through non-tax mechanisms; implement content-neutral advertising restrictions; and limit the quantity, type, and location of tobacco retailers.

#### Key accomplishments

More nimble than federal efforts, state and local governments are hubs of innovation for public health policy [113], particularly for policies to regulate the retail environment for tobacco. Specifically, states and localities have banned the sale of flavored tobacco, restricted promotions, limited where tobacco can be sold, and set minimum pack sizes and prices. Maine banned the sale of flavored tobacco products in 2007, followed by New York City in 2009 (enforcement began in 2010) and Chicago in 2013 the only jurisdiction to include menthol flavors. The Chicago policy applies only to sales near schools. Providence, Rhode Island (2012), and New York City (2013) prohibited price discounts, and Los Angeles (1991) imposed a content-neutral restriction on advertising to 10% of the total store window area. Finally, Boston banned the sale of tobacco products in pharmacies (2010), and San Francisco capped the number of sales permits for tobacco retailers and prohibited new retail licenses within 500 feet of schools and other tobacco retailers (2014).

#### Gaps in implementation

Innovative retail policies confront industry opposition and test legal boundaries, such as the first U.S. tobacco display ban [114] and a New York City mandate to display graphic warnings at the point of sale [115]. In a 2013 survey of state tobacco control programs, more programs mentioned legal support than funding as a resource necessary to promote retail regulation, and 78% of state tobacco control programs identified lack of political will as a barrier [116]. Additional capacity building is paramount. In a different survey of U.S. state and territory tobacco control program managers, all retail regulations received substantially lower readiness scores than other tobacco control policies [117]. For these reasons and more, innovative retail policies are not yet widespread.

#### Existing evidence and needed research

Tobacco industry and other opposition to retail policies frequently mention the lack of evidence that retail interventions will work [9,118,119]. For this reason, innovative state and local tobacco control requires a greater emphasis on solution-oriented research studies that are designed to inform policy and practice decisions [120,121]. Examples of solution-oriented research are experimental trials to test the likely impact of policy options, such as banning tobacco displays [122–124] and requiring graphic warnings at the point of sale [125]. Other study designs take advantage of natural experiments by comparing policy variation between countries [126,127] or data gathered pre- and post-implementation [128,129]. In addition, because the influence of retail policies on tobacco use is different from SHS policies, message framing research is needed to better understand how to communicate individual and population-level health benefits of retail policies to decision makers and the public. Despite accumulating evidence about the impact of retail marketing on cigarette smoking [130], public awareness about this important topic is lacking, and support for policy remedies is insufficient. Innovative state and local regulation is sometimes ahead of public opinion, and evidence suggests that public support for these policies increases after implementation [131–133]. To address this concern, some states have invested in media campaigns and advocacy efforts to increase awareness about tobacco retail marketing and its impact on youth [134–135]. Future research should compare the relative efficacy of framing policies as protections for youth, remedies for racial or economic injustice, and industry denormalization.

### Raise taxes on tobacco

#### Primary purpose

Tobacco taxes increase the price of tobacco products, often by an amount greater than the tax itself. Tobacco taxes are implemented as a fixed amount for a given quantity (i.e., excise tax) or as a percentage of the wholesale or retail price. In addition to reducing tobacco use, higher taxes generate additional revenues, which can be used to support comprehensive tobacco control programs.

#### Key accomplishments

In the United States, for example, inflation-adjusted cigarette prices more than tripled between 1980 and 2014, in large part due to a fourfold increase in inflation-adjusted average state cigarette taxes and in the federal cigarette tax [136]. During this time, the number of cigarettes consumed per capita decreased by nearly 70%, and the percentage of adults who smoke fell by half.

#### Gaps in implementation

As of July 1, 2016, all states tax cigarettes, but the level of taxes varies considerably, from a low of $0.17 in Missouri to a high of $4.35 in New York. Many localities also levy significant taxes, including Cook County, Illinois ($3.00), New York City ($1.50), and Chicago ($1.18). While cigarette excise taxes have increased significantly over time and across states, many other tobacco products are taxed at relatively low rates [137]. Currently, only the District of Columbia, Kansas, Louisiana, Minnesota, and North Carolina tax ENDS. There are some significant local taxes on ENDS, such as in Chicago. It is not clear how best to levy taxes across tobacco products and ENDS. For example, should differential taxes be used to reflect the continuum of risk of various products?

#### Existing evidence and needed research

A large body of literature, primarily focused on cigarettes, has demonstrated that significantly increasing tobacco product excise taxes is the single most effective policy for reducing tobacco use [138–141]. The resulting price increases reduce overall tobacco use, decrease tobacco use prevalence, spur many smokers to try to quit and lead some smokers to successful long-term cessation, keep former users from restarting, prevent youth from initiating tobacco use, and reduce consumption among those who continue to use [138]. Studies based on high-income countries generally find that a 10% price increase will reduce overall tobacco use by between 2.5% and 5% (4% on average). Youth are two to three times more price sensitive than adults, with the impact of higher prices particularly effective in preventing youth from moving from experimentation into regular daily use [138]. Likewise, estimates indicate that smoking rates among those in low SES groups are two to four times larger than those in high SES groups [142,143].

Higher tobacco taxes and prices also lead to other changes in behavior that may or may not have been fully anticipated [144–148]. For example, some users may switch to cheaper brands, whereas others may substitute to other tobacco products given changes in the relative prices of various products. Others may engage in efforts to avoid taxes (e.g. by buying online, from Indian reservations, or in nearby lower tax/price jurisdictions), whereas some may be more likely to take advantage of industry promotions that reduce tobacco product prices. Some may reduce spending on other goods/services to maintain expenditures on tobacco products [149–158].

Despite substantial research on cigarette taxes and prices, little is known about how taxes/ prices on other tobacco and nicotine products affect their prevalence, consumption, sales, initiation, and cessation; how different tax structures affect retail prices for these products; how tax/price changes for one tobacco product affect the demand for other tobacco products; and how different populations respond to tax increases. In the context of recent rapid increases in the use of ENDS and other new and emerging tobacco products, it is important to understand how the relative prices between combustible cigarettes and ENDS affect the initiation of use of ENDS and other new and emerging tobacco products; their impact on dual/poly use of those products and/or switching between those products; and their impact on the level of consumption of cigarettes and/or intentions to quit, quit attempts, and successful cessation of combustible cigarettes. In addition, in some states tax evasion undermines the impact of higher tobacco taxes and more research is needed to understand what policy and other interventions are effective at curbing tax evasion [159]. Finally, more research is needed to understand the impact of tobacco tax increases on household spending on other goods and services; how the burden of tobacco taxation change as tobacco taxes increase; and how the revenues generated by tobacco tax increases are used and their implications for the fairness of the tax.

### Dissemination and implementation opportunities for state and community tobacco control policy and program science

#### The challenge

As the preceding review illustrates, tobacco control remains a model for public health in how to establish scientific evidence supporting programs and policies implemented in states and communities. However, despite these successes, it still takes too long to translate tobacco control science into new policies and programs and too often proceeds in an ad hoc basis, resulting in a patchwork of policy coverage across the country. So, although it will continue to be important to expand the tobacco control science base, it is equally or even more important to ensure that this science gets delivered to the community in the form of evidence-based programs and policies. This is a critical challenge, as we recognize that translational and implementation sciences are just as important to decrease morbidity and mortality and increase public health as are new scientific discoveries [160].

#### The opportunity

**Figure 1:**
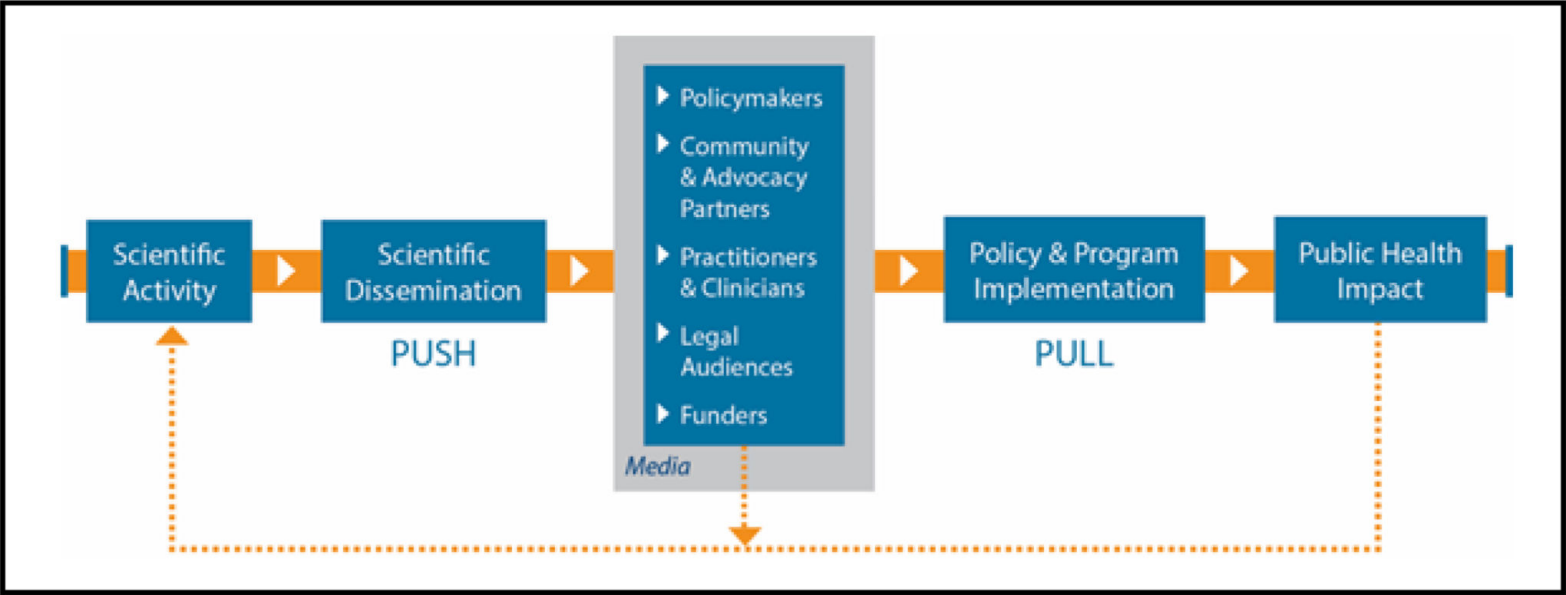
Tobacco control dissemination and implementation pipeline model.

Addressing this challenge requires (1) engaging in dissemination and implementation (D&I) activities more effectively, and (2) funding and conducting more dissemination science focusing on core state and community-level tobacco control issues. To the first point, it helps to understand what the barriers are for effective D&I. Figure 1 presents a pipeline model of the D&I process in tobacco control, suggesting how scientific information is translated and flows through a D&I process. D&I barriers are of two types: push barriers get in the way of the process of disseminating science to partners and communities, whereas pull barriers affect how communities are able to adopt and implement new programs and policies [161]. Push barriers include lack of training in dissemination skills, reliance on traditional or single modes of dissemination (e.g., ignoring social media), length of time to get scientific findings in systematic reviews and government reports (e.g., Surgeon General reports), and lack of timely dissemination planning [162,163]. Pull barriers, on the other hand, include not tailoring scientific information for particular audiences, failure to include tobacco control partners (who will be implementing the programs and policies) early enough in the research process, and lack of understanding of the policy development and implementation social system [161].

The role of tobacco control partners is critical in this process, as the pipeline model figure suggests. Failing to take into account the needs, perspectives, and goals of tobacco control policy makers, program managers, advocacy groups, clinicians, and legal experts will invariably slow down or completely stop the D&I process. Industry opposition to many of the new policy approaches being tested by communities and studied by tobacco policy scientists is predictable [164], and legal expertise is important to provide guidance on which evidence-based policies are more likely to survive legal and constitutional scrutiny. Finally, funders are also recognizing the importance of science-community partnerships for effective D&I. The current National Cancer Institute (NCI) State and Community Tobacco Control Research Initiative incorporates a number of partnership-enhancing mechanisms, including provision of internal funding for collaborative developmental research projects that strongly encourage outside partnerships, inclusion of community and advocacy partners at all scientific meetings, and the establishment of a Community Engagement Working Group.

Relatively little scientific attention has been paid to how evidence-based tobacco control policies and programs are disseminated to and implemented within community, clinical, and public health settings [165]. Fortunately, the general environment for D&I science is rapidly improving. Numerous theoretical frameworks exist for studying evidence-based D&I in public health [166,167]. NIH has supported D&I-focused requests for application, established the Dissemination & Implementation Research in Health study section, and sponsored an annual D&I research conference. New discoveries of the processes and characteristics of effective D&I in tobacco control would make it more likely that we could achieve our tobacco use reduction national goals.

## Conclusion and Research Recommendations

Spending on tobacco control interventions represents a wise public health investment. For example, many studies have shown that higher spending on state tobacco control programs is associated with decreases in youth, young adult, and adult smoking prevalence and per capita cigarette sales [35,71,168–171]. The Community Guide concludes that comprehensive tobacco control programs are cost-effective and that the averted health care costs from tobacco control exceed intervention costs [172]. Furthermore, there is abundant evidence of the cost-effectiveness of tobacco control policies [173]. Given the positive return on investment and the fact that tobacco use remains the leading cause of preventable premature death, a continued focus on the science and practice of state and community tobacco control is warranted. Specifically, we recommend a few particular salient and timely research priority areas.

In light of the evolving media landscape, it is critical to understand how people are exposed to and influenced by tobacco marketing and promotion as well as by antitobacco messages. Although evidence demonstrates that public education campaigns have been successful in reducing tobacco use among youth and adults, future research should investigate the effectiveness of alternative and nontraditional media channels in promoting or reducing tobacco use.

The use of ENDS has risen dramatically in recent years and now exceeds smoking prevalence among middle and high school students. In addition, approximately 16% of current smokers are current users of ENDS [174]. It is not yet clear how adult ENDS use is affecting the prevalence of adult smoking. Although ENDS likely are less harmful than combusted tobacco, they are not risk-free. Early research suggests that prolonged or repeated inhalation of propylene glycol may cause eye and respiratory irritation and may have adverse effects on pulmonary function [175], and exposure to e-cigarette aerosol promotes bacterial virulence and inflammation [176]. Although FDA has regulatory authority over ENDS, it will take time before FDA’s proposed and subsequent regulations affect ENDS use. In the meantime, there is an urgent need to understand how state and community tobacco control programs can effectively communicate and set policies to reduce population-level harm as the science around ENDS evolves. To do that, we need to understand how tobacco control policies influence use of ENDS (e.g., exclusive use or use with other tobacco products).

The legalization of marijuana for medical and/or recreational purposes raises new questions for tobacco control. How will marijuana legalization affect co-use of tobacco and marijuana and does it perpetuate existing disparities in cigar use? How common is vaping of hash oil among youth and adults and is it viewed as less harmful than smoking marijuana? Does the promotion of ENDS increase use of hash oil and vice versa?

Significant disparities persist in smoking prevalence related to income, education, race/ethnicity, health insurance coverage, and residence in rural areas [1]. The increased rates of smoking based on demographic variable such as these translate directly into disparities in lung cancer incidence and mortality [177]. State and community tobacco control efforts can reduce not only smoking, but overall lung cancer incidence [3,178]. Understanding the limitations of existing tobacco control strategies and identifying new approaches to reduce these disparities is a central challenge to further decreasing the health and economic burden of tobacco and reducing the population-level prevalence of smoking.

It is challenging for states and communities to adopt evidence-based tobacco control policies. Indeed, substantial declines in smoking prevalence over the past 40 years contribute to a public perception that other public health priorities warrant more attention. Research is needed about how best to frame tobacco control strategies about SHS interventions, tax and price policies, and the use of emerging technologies and multiple media channels to reduce disparities in tobacco use and to support implementation and sustainability of evidence-based programs and policies.

## Future Directions

Historically in the United States, states and communities have played crucial roles in testing and implementing tobacco prevention and control policies and programs and in designing and implementing mass media campaigns. Over the past several decades the NCI Tobacco Control Research Branch has funded research to study many of the various tobacco control policies and programs implemented around the country including studies of price and tax, smoke-free laws, state and local tobacco control programs, tobacco retail environment/density, media campaigns (pro- and counter-tobacco control), tobacco industry strategies, tobacco industry document research, and voluntary tobacco-related policies. Only a few of the U.S. states invest their own dollars specifically in tobacco control policy research (e.g., California and Minnesota), which means that, for the most part, states rely on the coordinated scientific efforts from agencies such as NCI, and they rely on the technical and community grant support from service agencies, such as CDC, to help guide their local efforts. Working together with new tools, technologies, and data sources, the scientific and service agency partnerships help foster novel lines of inquiry and make rapid scientific progress for tobacco control program and policy science a reality. These are exciting times for the promise of advancements in state and community tobacco control program and policy science, and we also may see eventual synergies between tobacco control efforts at the state and local levels with forthcoming federal regulations [179]. Further successes in preventing, treating, and controlling tobacco use and remedying the disparities in tobacco use and tobacco-related disease will require innovative and targeted efforts for state and community tobacco control program and policy science in the future.
